# Longitudinal Analysis of Nut-Inclusive Diets and Body Mass Index Among Overweight and Obese African American Women Living in Rural Alabama and Mississippi, 2011–2013

**DOI:** 10.5888/pcd14.160595

**Published:** 2017-09-21

**Authors:** Samara R. Sterling, Brenda Bertrand, Suzanne Judd, Tiffany L. Carson, Paula Chandler-Laney, Monica L. Baskin

**Affiliations:** 1 Department of Nutrition Science, School of Health Professions, University of Alabama at Birmingham, Birmingham, Alabama; 2 Department of Biostatistics, School of Public Health, University of Alabama at Birmingham, Birmingham, Alabama; 3 Division of Preventive Medicine, School of Medicine and Comprehensive Cancer Center, University of Alabama at Birmingham, Birmingham, Alabama

## Abstract

**Introduction:**

Nuts, when eaten alongside other nutritionally rich foods, may decrease obesity and related chronic disease risks, which are high among African American women in the rural South. We monitored changes in nut intake, other obesity-related foods (fruits, vegetables, red or processed meats, added sugars), and body mass index (BMI) over a 2-year weight loss intervention among 383 overweight and obese African American women in rural Alabama and Mississippi.

**Methods:**

Two dietary recalls were administered at 4 points over 24 months. Mann–Whitney tests compared differences in median food group intake between nut consumers and non-nut consumers, and *t* tests identified BMI differences between groups. Mixed linear models tested the relationship between nut intake and intake of the select food groups, and between nut intake and BMI over time.

**Results:**

Overall nut consumers ate more fruits and vegetables and less red meat than non-nut consumers. Nut consumers had lower BMI values than non-nut consumers. Weight loss by the end of the intervention was significant for nut consumers but not for non-nut consumers, even after accounting for kilocalorie consumption and physical activity engagement.

**Conclusion:**

Nut consumption is associated with consumption of other nutritionally rich foods and lower BMI among African American women in rural Alabama and Mississippi. Future interventions should target increasing daily nut intake, decreasing added sugar intake, and identifying strategies to encourage positive dietary changes to continue after an intervention.

## Introduction

African American women in the rural southeastern United States have the highest rates of obesity and obesity-related diseases in the country (1,2). Eighty percent of African American women in the United States are either overweight or obese (3). This disparity may be the result of various influences (eg, environmental, cultural, behavioral) associated with low diet quality, resulting in high rates of obesity and chronic diseases (4,5).

African Americans in the rural South tend to consume a traditional “Southern” diet that contains large amounts of red or processed meats, salty snacks, and added sugar (6,7), which increase obesity and chronic disease risks (6). However, although these foods are in the diet, many protective plant foods such as collard greens, apples, green beans, and nuts are abundant in southern regions and are included in Southern cuisine (8–12). Nuts in particular may facilitate weight loss, and dietary patterns that include them generally result in lower rates of obesity and chronic disease risks (13–16). What is unknown is whether the consumption of nuts alongside other protective foods over time enhances synergistic health benefits (17) or whether the incorporation of nuts into a traditional Southern diet increases obesity risk. Weight loss interventions in this community are challenging because of high attrition rates, low weight loss maintenance, and failure to tailor dietary recommendations on the basis of foods already in the diet (18).

The objective of this study was to examine the longitudinal relationship between nut intake and other healthful foods (eg, fruits and vegetables) and foods whose intake is related to obesity and chronic disease (eg, red or processed meats, added sugars) in a 2-year weight-loss intervention among African American women in rural Alabama and Mississippi. We examined changes in body mass index (BMI, measured as weight in kg divided by height in m^2^) between nut and non-nut consumers. We hypothesized that nut consumers would eat more fruits and vegetables, have lower BMI values, and lose more weight than non-nut consumers.

## Methods

We used secondary data from the Deep South Network for Cancer Control (DSN). DSN is an ongoing collaboration among university researchers, public health practitioners, and volunteers who live and work in target communities. From its inception in 2000, the aim of DSN has been to eliminate cancer disparities, particularly in rural communities of Alabama and Mississippi (19). For this study, analyses were performed on a subgroup of 383 overweight and obese African American women who participated in a 2-year DSN weight loss intervention from 2011 through 2013.

Eight rural counties, evenly distributed between Alabama and Mississippi, were selected for the intervention. Participants lived or worked in one of these counties. Selected counties have limited access to health care and high poverty and cancer rates (19). Half of the counties, evenly distributed between states, received the group weight loss intervention, and the other counties received community strategies along with the weight loss intervention. The community strategies included grants to fund farmers’ markets and produce stands in the community. The research protocol was approved by the institutional review board at the University of Alabama at Birmingham, and all participants provided written informed consent.

### Recruitment and exclusion criteria

Recruitment for the parent study was conducted from January 2011 through September 2013 by study staff who lived in the communities of study. Participants were recruited through networking, word of mouth, and announcements in churches, health departments, schools, and other local facilities. People who were eligible self-identified as African American; lived, worked, or attended school in a participating community; were aged 30 to 70 years; had a measured BMI of 25 or greater; reported no history of weight loss surgery, eating disorder, recent cardiac event, or mobility impairment; and reported being a nonsmoker. Women were excluded at the baseline assessment if they had uncontrolled blood pressure (systolic blood pressure ≥160 mm Hg or diastolic blood pressure ≥100 mm Hg) or fasting blood glucose of 126 mg/dL or higher. Data from participants of the parent study who provided at least 2 dietary recalls over the study period (n = 383) were used for this study (Figure). 

**Figure:**
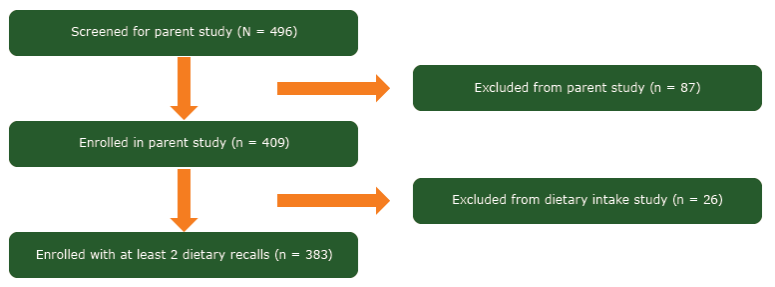
Study cohort enrollment diagram for 383 overweight and obese African American women enrolled in a weight loss intervention in rural Alabama and Mississippi, 2011–2013.

### Demographic information and body mass index

Participants completed baseline demographic surveys that included questions about their age, employment status, annual household income, education level, and marital status. Trained staff members measured height and weight at baseline and at 6, 12, and 24 months. Height and weight were measured with a portable stadiometer (SECA 2-in-1 model no. 8761321004; seca GmBh & Co KG). Height was measured to the nearest 0.1 cm, and weight to the nearest 0.1 kg with light clothing and without shoes. BMI was calculated from height and weight measurements (20).

### Intervention

The weight loss intervention was conducted during a 2-year period and included the first 6 months of intensive evidence-based education about obesity and chronic disease prevention as the initial study phase. By county, participants met once per week and were educated by trained volunteers about the role of healthy habits, including diet and regular physical activity, in promoting weight loss and preventing chronic disease. Participants were introduced to various weight loss strategies including reducing daily calorie intake, adhering to an exercise regimen, and preparing healthy meals. The weekly intervention meetings included discussions about participant successes and challenges to achieving their health goals, and participants received group support. The intensity of the intervention was gradually decreased, and in the following 6 months groups met twice per month (3 months), then monthly (3 months) to discuss maintenance of healthy habits learned in the program. During the second year (maintenance phase), group meetings were discontinued, and participants received monthly telephone calls from lay peer coaches to discuss maintenance of the healthy habits.

### Dietary intake data collection

Dietary data were collected by using the Automated Self-Administered 24-Hour Dietary Recall (ASA24). ASA24 is a web-based tool that guides participants through recording all foods eaten on a previous day (21). ASA24 uses the Automated Multiple Pass Method (AMPM), which is an evidence-based approach intended to improve the accuracy of food intake recording in 24-hour dietary recalls (21,22). AMPM uses multiple probes throughout the recall to prompt users to remember all foods eaten in the previous day, including commonly forgotten foods. Because of limited computer access, participants completed the recalls with trained staff members by telephone and in person.

Dietary information was collected at baseline and at 6, 12, and 24 months. Each answer was entered into ASA24 at the time of interview. At each point, trained staff collected 1 weekday recall in person and 1 weekend recall by telephone. The rationale for 1 in-person and 1 telephone call was logistical. Because research study staff traveled to local communities (up to 4 hours away) to collect a weekday recall, capturing the weekend recall via telephone eliminated the need for participants and staff members to return to the assessment location for a single assessment. Visual cue cards were provided to participants preceding interviews to help them identify common household measurements, such as teaspoons and cups. The validity of the telephone method of administration for recalls has been established. Research indicates that the estimation of energy intakes was as effective in the telephone method as in the in-person method (22). Dietary recalls were collected from 383 participants during the 2-year study period.

### Dietary intake nutrient analysis and physical activity

The ASA24 database was linked with the MyPyramid Equivalents Database (MPED) from the US Department of Agriculture. MPED standardizes the quantities of reported food groups (23). In MPED, protein foods, such as red meat and processed meats, were measured in ounces. Nuts, including peanuts, tree nuts, and seeds, were measured in ounce equivalents (oz eq), where 0.5 oz of nuts is nutritionally equivalent to 1 oz of lean meat. Fruits and vegetables were measured in cup equivalents (cup eq) of whole fruit (not including juice) and total vegetables, and added sugars were calculated in teaspoon equivalents (tsp eq).

Nut consumers were identified by points in time and overall. At each point, nut consumers were identified as participants who consumed nuts, whereas non-nut consumers were participants who did not consume nuts at that point. Overall nut consumers were defined as participants who ate nuts during at least 1 of the 4 points, whereas non-nut consumers did not.

Participants completed surveys that asked 2 questions about the number of days per week they engaged in physical activity (moderate and/or vigorous physical activity) at each of the 4 points. Moderate physical activity was defined as “physical activity that causes some increase in breathing or heart rate” (eg, brisk walking, bicycling, vacuuming, gardening). Vigorous activity was defined as “physical activity that causes large increase in breathing or heart rate” (eg, running, aerobics, heavy yard work) (24).

### Statistical methods

Four dietary recall points at 6-month intervals over a 24-month period were examined to reflect changes in dietary intake over time. Participation rate and the proportion of nut to non-nut consumers overall and at each point were calculated. Participants who completed dietary recalls for at least 1 of the 4 points were included in the analysis. The linear mixed models accounted for missing data and drop-out patterns by adjusting for random variables and time-varying covariates, such that an unequal number of observations across participants did not negatively affect the results (25).

The food groups and components that were examined included nuts, fruits, vegetables, red meat, processed meat, and added sugars. These food groups were chosen on the basis of their association with obesity, obesity-related chronic diseases, or both (15,26–28). To account for underlying data distribution, we calculated median intake of nuts and of these food groups or components of interest at each point. We used Mann–Whitney tests to compare differences in median food group intake at each point between groups. After the Johnson SI transformation was applied to BMI, *t* tests were used to examine differences in mean BMI between groups at each point.

Mixed linear models were used to examine the relationship between nut intake and the intake of the select food groups and components over time using a factorial design. In this model, nut intake at baseline and at 6, 12, and 24 months (points 1, 2, 3, and 4, respectively) was the independent variable and the food groups and components at baseline and at 6, 12, and 24 months were the dependent variables. The model also accounted for the variability within and between participants. The relationship between nut intake and BMI over time was also identified by using interaction testing. Both mixed models were adjusted for age, annual household income, education level, and mean daily kilocalorie intake. The relationship between nut intake and BMI was further adjusted for number of days per week of moderate and vigorous physical activity reported. The slopes were tested to determine the presence of a significant change in the association between nut intake and intake of select food groups and components and BMI over time. All statistical tests were conducted using JMP Pro 12 (SAS Institute, Inc), and *P* values of .05 or less were considered significant.

## Results

Seventy percent of the 383 participants were overall nut consumers, and approximately 38% of participants consumed nuts at each point (Table 1). Nut consumers and non-nut consumers were similar at baseline in BMI, age, annual household income, and education level (Table 2).

**Table 1:** Dietary Recall^a^ Participation Among 383 Overweight or Obese African American Female Nut Consumers and Non-Nut Consumers in Rural Alabama and Mississippi, 2011–2013

Time	Participation Rate^b^	Nut Consumers^c^	Non-Nut Consumers^d^
	Proportion (%)		
Overall	383 of 383 (100)	267 of 383 (70)	116 of 383 (30)
Baseline	382 of 383 (99.7)	160 of 382 (42)	222 of 382 (58)
6 Months	328 of 383 (86)	119 of 328 (36)	209 of 328 (64)
12 Months	287 of 383 (75)	116 of 287 (40)	171 of 287 (60)
24 Months	232 of 383 (61)	79 of 232 (34)	153 of 232 (66)

a Dietary recalls were administered using the web-based Automated Self-Administered 24-Hour (ASA24) recall system.

b Women who participated in the dietary recall analysis portion of an intervention of the Deep South Network for Cancer Control.

c Participants were classified as nut consumers if they reported consuming nuts on at least 1 of the 4 dietary recall points.

d Participants were classified as non-nut consumers if they did not report consuming nuts on at least 1 of the 4 dietary recall points.

**Table 2:** Baseline Demographic Characteristics of 383 Overweight or Obese African American Female Weight Loss Participants in Rural Alabama and Mississippi, 2011–2013

Baseline Description^a^	Total	Nut Consumers	Non-Nut Consumers
BMI, mean (SD), kg/m^2^	38.6 (8.1)	38.0 (7.5)	39.1 (8.6)
Age, mean (SD), y	47 (10)	47 (11)	46 (10)
**Annual household income, $**			
<10,000	73 (19)	23 (15)	50 (23)
10,000–19,999	87 (23)	41 (26)	46 (21)
20,000–29,999	80 (21)	33 (21)	47 (22)
30,000–39,999	57 (15)	22 (14)	35 (16)
40,000–49,999	32 (9)	18 (11)	14 (6)
≥50,000	36 (10)	18 (11)	18 (8)
Don’t know/unsure	11 (3)	3 (2)	8 (4)
Missing	6 (1)	2 (1)	4 (2)
**Education level**			
Less than high school	22 (6)	5 (3)	17 (8)
High school graduate/GED	129 (34)	51 (32)	78 (36)
Some post high school	71 (19)	31 (20)	40 (18)
College graduate or more	152 (41)	70 (45)	82 (38)
Don’t know/unsure	1 (0)	0 (0)	1 (0)
Missing	7 (2)	3 (2)	4 (2)

Abbreviations: BMI, body mass index; GED, general educational development; SD, standard deviation.

a There were no significant differences in BMI, age, income, or education between nut consumers and non-nut consumers at baseline (*P* = .19, *P* = .32, *P* = .27, and *P* = .06, respectively). Values are presented as no. (%), unless otherwise indicated.

### Food consumption differences between nut and non-nut consumers

Overall nut consumers had significantly higher intakes of fruits and vegetables and lower intakes of red meat than did non-nut consumers (Table 3). However, nut consumers and non-nut consumers reported similar dietary trends at baseline and at 24 months (Table 3). At 24 months, nut consumers consumed significantly less median daily sugar than they did at baseline (4.7 less tsp eq/d, *P* < .001; 1.6 less tsp eq/1,000 kcal/d, *P* = .004). When accounting for kilocalories consumed, however, there were no significant differences in median nut, fruit, vegetable, or red meat intake between baseline and 24 months. Results were similar for non-nut consumers, who also reported consuming less added sugar at 24 months than they did at baseline (2.7 less tsp eq/d, *P* < .001; 0.8 less tsp eq/1,000 kcal/d, *P* = .01). They also reported significantly lower processed meat intake between baseline and 24 months (*P* = .02). However, there was no change in median fruit, vegetable, or red meat intake between baseline and 24 months. There was no difference in added sugar intake between nut consumers and non-nut consumers when comparing baseline and 24 months (*P* = .30).

**Table 3:** Difference in Food Group/Component Consumption and BMI Between 383 Overweight and Obese African American Nut Consumers and Non-Consumers in Rural Alabama and Mississippi, at Baseline, 6 Months, 12 Months, and 24 Months, 2011–2013

Food Group/Component	All Participants	Nut Consumers	Non-Nut Consumers	*P* Value^a^
	Median (25th–75th Percentile)/Median Per 1,000 kcal			
**Nuts, oz equivalent**				
Overall	—	0.4 (0.1–1.2)/0.3	—	—
Baseline	—	0.4 (0.1–1.0)/0.3	—	—
6 Months	—	0.5 (0.2–1.5)/0.4	—	—
12 Months	—	0.4 (0.1–1.3)/0.3	—	—
24 Months	—	0.5 (0.2–1.0)/0.3	—	—
Adjusted *P* value (per 1,000 kcal)^b^	—	.19	—	—
**Whole fruit, cup equivalent**				
Overall	0.4 (0–1.0)/0.3	0.4 (0–1.1)/0.4	0.2 (0–0.9)/0.2	<.001
Baseline	0.2 (0–0.9)/0.2	0.6 (0.1–1.0)0.3	0.1 (0–0.7)/0.1	<.001
6 Months	0.5 (0–1.4)/0.5	0.6 (0.2–1.6)/0.5	0.5 (0–1.3)/0.4	.09
12 Months	0.4 (0–1.1)/0.3	0.5 (0.1–1.4)/0.4	0.4 (0–1.0)/0.3	.01
24 Months	0.3 (0–1.0)/0.2	0.4 (0–1.3)/0.4	0.2 (0–0.9)/0.2	.17
Adjusted *P* value (per 1,000 kcal)^b^	.005	.009	.20	.22^c^
**Vegetables, cup equivalent**				
Overall	1.1 (0.7–1.6)/0.8	1.2 (0.7–1.7)/0.8	1.0 (0.6–1.5)/0.8	<.001
Baseline	1.1 (0.7–1.6)/0.7	1.2 (0.8–1.7)/0.7	1.0 (0.7–1.5)/0.7	.04
6 Months	1.2 (0.7–1.8)/0.9	1.3 (0.8–1.8)/0.9	1.2 (0.7–1.8)/0.9	.25
12 Months	1.1 (0.7–1.6)/0.5	1.2 (0.7–1.6)/0.8	1.1 (0.6–1.6)/0.8	.44
24 Months	1.1 (0.6–1.6)/0.8	1.1 (0.6–1.6)/0.8	1.0 (0.6–1.5)/0.8	.46
Adjusted *P* value (per 1,000 kcal)^b^	.009	.01	.38	.11^c^
**Red meat, oz**				
Overall	0.7 (0–1.8)/0.5	0.6 (0–1.7)/0.4	0.9 (0–2.0)/0.6	.01
Baseline	0.9 (0.1–2.0)/0.6	0.9 (0–2.0)/0.4	0.1 (0.2–2.0)/0.7	.07
6 Months	0.5 (0–1.5)/0.3	0.4 (0–1.2)/0.2	0.5 (0–1.8)/0.4	.11
12 Months	0.6 (0–1.9)/0.5	0.6 (0–1.7)/0.5	0.6 (0–1.9)/0.5	.28
24 Months	0.6 (0–1.8)/0.6	0.3 (0–1.5)/0.3	0.7 (0–1.8)/0.6	.41
Adjusted *P* value (per 1,000 kcal)^b^	.83	.43	.82	.43^c^
**Processed meat, oz**				
Overall	0.2 (0–0.9)/0.2	0.2 (0–0.9)/0.2	0.3 (0–0.9)/0.2	.59
Baseline	0.4 (0–1.1)/0.3	0.4 (0–1.0)/0.2	0.4 (0–1.1)/0.3	.43
6 Months	0.2 (0–0.8)/0.1	0 (0–0.7)/0	0.3 (0–0.9)/0.2	.09
12 Months	0.2 (0–0.9)/0.1	0.3 (0–0.9)/0.2	0.2 (0–0.9)/0.1	.77
24 Months	0 (0–0.8)/0	0 (0–0.9)/0	0.04 (0–0.8)/0.04	.98
Adjusted *P* value (per 1,000 kcal)^b^	.18	.71	.07	.14^c^
**Added sugar, tsp equivalent**				
Overall	9.4 (4.9–14.7)/7.2	9.6 (4.9–14.9)/7.4	9.0 (4.5–14.5)/7.0	.16
Baseline	12.0 (7.5–17.6)/8.1	13.0 (8.7–20.0)/8.2	10.8 (6.6–16.7)/7.8	.005
6 Months	8.4 (3.7–13.4)/6.3	9.5 (5.8–14.0)/6.7	7.3 (2.9–13.0)/6.1	.003
12 Months	8.7 (4.3–14.3)/6.6	8.8 (4.4–14.8)/6.6	8.3 (4.2–14.1)/6.7	.48
24 Months	8.2 (4.4–12.8)/6.8	8.3 (4.4–13.2)/6.6	8.1 (4.3–12.4)/7.0	.51
Adjusted *P* value (per 1,000 kcal)^b^	<.001	.004	.01	.57^c^
**Body mass index, kg/m^2^, mean (SD)**				
Overall	38 (8)	37 (7)	40 (9)	<.001^d^
Baseline	39 (8)	38 (7)	39 (9)	.33^d^
6 Months	37 (8)	36 (7)	38 (8)	.25^d^
12 Months	37 (8)	36 (7)	38 (8)	.009^d^
24 Months	38 (8)	37 (7)	38 (8)	.07^d^

Abbreviations: — , does not apply; BMI, body mass index; SD, standard deviation.

a *P* values were determined by Mann–Whitney tests and compare differences in food intake between nut and non-nut consumers at each of the 4 time points.

b *P* values were determined by linear mixed models and compare changes in food group intake over time in all participants, nut consumers, and non-nut consumers while adjusting for daily kilocalorie consumption.

c *P* values were determined by linear mixed models and compare differences in dietary changes over time between nut and non-nut consumers while adjusting for daily kilocalorie consumption.

d *P* values were determined by *t* tests and compare BMI differences between nut and non-nut consumers at each time point.

Similar results were observed when conducting longitudinal analyses of food group and component intake over time in nut consumers and non-nut consumers separately. When adjusting for kilocalories consumed, there were no changes in red or processed meat intake over time in either group, and non-nut consumers did not change their fruit or vegetable intake over time. However, nut consumers increased their fruit (*P* = .009) and vegetable (*P* = .01) intake over time. Furthermore, the decrease in daily added sugar intake per 1,000 kcal over time was significant for both nut consumers (*P* = .004) and non-nut consumers (*P* = .01). The change from baseline to 24 months in total daily added sugar intake (*P* = .07) and daily added sugar intake per 1,000 kilocalories (*P* = .57) did not differ between groups enough to attain significance. When adjusting for age, annual household income, and education level, both nut consumers and non-nut consumers significantly decreased their mean daily kilocalorie consumption throughout the intervention (*P* < .001 for both groups).

### Longitudinal relationship between nut intake and BMI

Overall nut consumers had significantly lower BMI values than did non-nut consumers over the study period (*P* < .001, Table 3) and at each point (baseline, *P* = .04; 6 months, *P* = .009; 12 months, *P* = .003; 24 months, *P* < .001). Nut consumers at each point had lower BMI values than did non-nut consumers (Table 3). In the longitudinal analysis, BMI values of all participants decreased over the 2-year period (*P* = .002), although this result was significant in nut consumers (*P* = .01) but not in non-nut consumers (*P* = .63). For nut consumers, this finding remained after the model was adjusted for age, annual household income, and education level (*P* < .001) and for number of days per week of exercise (*P* < .001) and mean daily kilocalories consumed (*P* = .05).

## Discussion

We monitored changes in dietary intake over a 2-year weight loss intervention in 383 overweight and obese African American women in rural Alabama and Mississippi. Specifically, we wanted to identify whether or not nut consumption was accompanied by a higher intake of other protective plant foods and the association between nut intake and BMI.

Our main finding was that, even when adjusting for confounders, nut consumption was consistently associated with lower BMI over time. A possible explanation for this is that nut consumers ate more plant foods and less red meat than did non-nut consumers. Diets that emphasize nuts, fruits, and vegetables and limit red meat are protective against obesity and chronic diseases (13). Even though median nut intake among nut consumers remained stable over the intervention, the overall diet of nut consumers may have contributed to weight management. One previous study found lower BMI values in individuals who consumed nuts long-term compared with those who did not, despite the amount consumed (29). These results coincide with our findings and suggest that a small amount of nut intake may be associated with significant weight management benefits if consumed over a long period. Nut consumers also significantly reduced their added sugar intake from baseline to 24 months, which may have further assisted with weight loss (28).

An area of concern observed in this study was that some of the positive dietary behaviors noted in both nut consumers and non-nut consumers did not persist to the end of 24 months. It is common for African Americans in the South to fall short of federal recommendations for intake of fruits and vegetables (30). Previous lifestyle interventions in African American communities have described similar challenges in participants maintaining healthy behaviors after an intervention (18). Because food choices in African American communities are closely linked to cultural traditions, adhering to a pattern that excludes cultural foods that are less nutritious may be challenging, even for those who desire to lose weight (4). Positive dietary changes must remain after an intervention period for participants to continue to experience the health benefits gained during the intervention.

A strength of this study was that it used a large sample size, which was also homogeneous in race, weight status, geographic location, and sex. Therefore, results provide a reliable examination of nut-inclusive diets among overweight and obese African American women who participated in the DSN study in the rural South. One weakness included reliance on self-reported data, such as dietary intake and physical activity. However, these were the most economically feasible approaches for this study. Caution should be used when generalizing the findings to women of other races, age groups, and regions of the country.

The impact of future weight loss interventions in this community could be enhanced by encouraging increased nut intake and decreased added sugar intake and by introducing low-calorie substitutions for popular Southern foods such as red and processed meats. Interventions may be able to encourage long-term dietary changes by focusing on maintaining a group or family support system after the intervention. Participants may be trained as leaders to teach community members the material learned in the intervention. An ongoing system of community involvement may encourage more widespread and long-term positive changes after the intervention. Future studies may also examine the effect of nut consumption on metabolic processes among women in this population.
